# Khat use and associated factors during pregnancy in eastern Ethiopia: A community-based cross-sectional study

**DOI:** 10.3389/fgwh.2022.941300

**Published:** 2022-12-02

**Authors:** Tadesse Misgana, Dejene Tesfaye, Daniel Alemu, Berhe Gebremichael, Dawit Tamiru, Mandaras Tariku, Adisu Birhanu Weldesenbet, Merga Dheresa

**Affiliations:** ^1^ Department of Psychiatry, College of Health and Medical Sciences, Haramaya University, Harar, Ethiopia; ^2^ School of Public Health, College of Health and Medical Sciences, Haramaya University, Harar, Ethiopia; ^3^ Department of Midwifery, College of Health and Medical Sciences, Haramaya University, Harar, Ethiopia; ^4^ School of Nursing and Midwifery, College of Health and Medical Sciences, Haramaya University, Harar, Ethiopia

**Keywords:** khat use, pregnant women, substance use, associated factors, eastern Ethiopia

## Abstract

**Introduction:**

Women of reproductive age are increasingly using khat. The use of khat is associated with prelabor rupture of membranes, anemia among pregnant women, and other problems related to motherhood and infanthood. Most of the previous studies performed at the facility level revealed that different factors were associated with khat use among pregnant women. Lower educational status, low wealth index, and the age of the mother were the factors significantly associated with maternal khat use. Partner substance use also has a significant association with maternal khat use. However, there is limited information about khat use and its associated factors among pregnant women in the study area.

**Objective:**

This study aims at assessing the prevalence of khat use and associated factors among pregnant women in Kersa and Haramaya Health and Demographic Surveillance System Sites, eastern Ethiopia.

**Methods:**

A community-based cross-sectional study design was employed among randomly selected 1,015 pregnant women from an open cohort from Kersa and Haramaya Health and Demographic Surveillance System Sites in Ethiopia. Data were collected through face-to-face interviews from January 30 to April 30, 2021, using Open Data Kit (ODK) software and analyzed using SPSS v-26. Descriptive statistics were used to summarize the characteristics of pregnant women. Factors associated with khat use were identified by bivariate and multivariable logistic regression analyses; an adjusted odds ratio (AOR) with a 95% confidence interval (CI) was estimated. Statistical significance was declared at *p* < 0.05.

**Results:**

The prevalence of khat use among pregnant women was 15.5% (95% CI, 13.3–17.7). Age of the pregnant women; being in the age group between 25 and 35 years (AOR = 2.27, 95% CI, 1.33–4.89) and 35 years and greater (AOR = 2.33, 95% CI, 1.29–4.20); having a chronic medical illness (AOR = 3.28, 95% CI, 1.27–8.48); and having a history of abortion (AOR = 2.87 95% CI, 1.73–4.76) significantly increased the likelihood of khat use among pregnant women.

**Conclusion:**

The current study revealed a relatively high magnitude of khat use in pregnant women as compared with previous studies. The age of the pregnant women, history of medical illness, and history of abortion were significantly associated with khat use during pregnancy.

## Introduction

Khat (*Catha edulis*) is an evergreen plant that is widely used in the Middle East, Somalia, East Africa, and Ethiopia (1, 2). Cathinone is the most important chemical found in young leaves and shoots of khat. It also contains cathine, nor-ephedrine, and other chemicals (3). Khat is believed to have the same effect as amphetamine, a stimulant of the central nervous system (4). Acute oral administration of khat offers a sense of euphoria, cheerfulness, relief from fatigue, increased energy levels, the ability to communicate easily, the capacity to associate ideas, and improved self-confidence (5). However, these symptoms are typically followed by discomforts including depression, anxiety, and insomnia (1).

Khat chewing poses many health-related problems. Khat chewing among reproductive-age women is on the rise, and it is a risk factor for serious reproductive health problems among pregnant women. These problems include intrauterine growth retardation, low birth weight (LBW), and increased infant mortality (2). It also is a risk factor for lower lipoid level, sexual impotence, and inhibition of uteroplacental blood flow, which can lead to a teratogenic effect and the impairment of fetal growth (6). Hence, among pregnant women, consumption of khat affects the growth of the fetus by inhibiting uteroplacental blood flow and, consequently, impairs fetal growth (2).

Complex physiological changes occur during pregnancy and result in the impairment of sleep and memory. The adverse effects of khat on memory and sleep may further aggravate those impairments (7). Problematic khat use is a possible risk factor for harmful use of other psychoactive substances such as alcohol, and it was observed that one of the risk factors for engaging in risky sexual behavior was pushed by the effect of khat after chewing (8).

A national longitudinal survey conducted in the United States showed that women with a history of abortion were reported to use different types of substances (9, 10). In another national population survey carried out in Yemen, about 40.7% of women surveyed reported chewing khat while pregnant during the previous 5 years before the survey. This study also revealed that old age, no education, rural residence, living in mountainous regions, and low wealth were significant risk factors for chewing khat (5). A study designed to determine the psychosocial correlates of substance use among women attending antenatal services at a tertiary hospital in southwestern Nigeria revealed that the presence of medical conditions was associated with substance use among pregnant women (11).

A study conducted in eastern Ethiopia revealed that khat chewing is associated with prelabor rupture of membranes among pregnant women (12). Another study performed in rural communities in eastern Ethiopia indicated that khat chewing was associated with anemia among pregnant women (13). Cross-sectional studies conducted at primary healthcare centers in Ethiopia revealed that the prevalence of khat chewing ranged from 9.9% to 35.8% of pregnant women chewing khat during the current pregnancy (14, 15).

Khat use increased with increasing age, remaining constant after age 35, having a child, having a lower educational level, and not pertaining to the lowest wealth index category. Not being in a marital relationship with the most recent sex partner and protestant religion were protective factors (16). The outcome of the multivariable analysis showed that partner khat use, alcohol use, and mental distress were factors significantly associated with current khat use (15).

Despite the high prevalence of khat use during pregnancy, the focus of most studies has been to assess the magnitude of khat use among the general population and college students, but the magnitude of khat use among pregnant women and its determinant factors are not well addressed in Ethiopia. As per the authors’ knowledge, there is no empirical evidence reporting on the magnitude, pattern, and associated factors of khat use during pregnancy in the study area. Therefore, this study aims at filling this gap by assessing the magnitude and associating factors of khat use among pregnant women in eastern Ethiopia. The findings of this study can also help planners and decision-makers by showing the magnitude and pattern of khat chewing during pregnancy. Furthermore, the findings of this study will be used as baseline information for further studies.

## Methods and materials

### Study setting, period, and study design

This study has employed a community-based cross-sectional study design with the quantitative method. It was conducted in Kersa and Haramaya Health and Demographic Surveillance Sites (HDSS) from January 30 to April 30, 2021. Kersa HDSS is in Kersa District, Oromia Regional State, and eastern Ethiopia. There are 35 rural subdistricts (called kebeles) and three small-town kebeles. According to the 2007 national census, the district has a total population of 172,626 of whom 6.9% are urban dwellers. The Kersa HDSS covers 24 of the 38 kebeles, with 4 health centers and 10 health posts. There are 18 elementary, 2 secondary, 1 preparatory, and 2 religious schools in the HDSS area, as well as 134 mosques, 8 churches, and 6 farmers’ training stations. Most of the inhabitants are farmers, with a minority working in small trade, government posts, or casual laborers (17). Haramaya HDSS is a new site, which was established in 2018. It covers 12 rural kebeles. At baseline, the total number of households in the HDSS was 17,461, and the total population was 99,898 (51,259 males and 48,639 females), of whom 23.86% were women of reproductive age (18).

### Characteristics of the population

All pregnant women who were living in Kersa and Haramaya HDSS sites were the source population, and all pregnant women who were in the selected kebeles from January 30 to April 30, 2021, were included in the study. Pregnant women who were not able to communicate due to serious medical illnesses during the study period were excluded from the study.

### Data collection measurements

Semistructured and structured questionnaires were deployed to gather data on participants’ khat use during the current pregnancy, sociodemographic variables such as age, marital status, religion, ethnicity, educational status, occupation, and wealth index. Other factors include reproductive, obstetric, and gynecological characteristics such as gravidity, parity, gestational age, history of abortion, antenatal care follow-up, interval between last delivery and last normal menstrual period, history of gynecological problem, history of gynecological operation; and psychosocial and other substance use characteristics, such as intimate partner violence (IPV), alcohol use (ever and current), tobacco (ever and current), and other substances.

Current khat use was assessed based on questions developed from different pieces of literature (15, 19). A study participant who reported chewing khat during the current pregnancy was considered a current khat chewer. Ever-use of substances was assessed based on the questions developed from various kinds of literature too (15, 19). A study participant who answered “yes” to a question that asked about lifetime use of khat was considered an ever-user of khat. Common mental disorder (CMD) was assessed by using the self-reporting questionnaire (SRQ-20). The SRQ-20 is composed of 20 yes/no items asking about the experience of depression, anxiety, panic, and somatic symptoms in the preceding 30 days. For this study, the total score was dichotomized (SRQ-20 < 6 vs. SRQ ≥ 6), and those who scored SRQ ≥ 6 indicated having CMD (20). Household economic status was assessed by using questions adapted and modified from the Ethiopian Demographic and Health Survey (EDHS) of 2016 (21). The experience of IPV was assessed using the World Health Organization (WHO) multicountry study questionnaire constituting psychological, physical, and sexually violent acts often accompanied by controlling behavior where a single positive answer indicated the presence of violence (22).

### Sample techniques/procedures

The sample size was estimated using a single population proportion formula, with the assumptions that, the corresponding standard value (*Z_α_*_/2_) at 95% confidence level = 1.96, 4% of margin of error, =4%, design effect (deff) = 1.5, the proportion of khat chewing (*p*) among pregnant women from the previous study conducted in Butajira (14), southern Ethiopia = 35.8%. Then, the result gave a sample size of 828. By considering a 10%, nonresponse rate the final sample size gave 911.

For the factors associated with current khat use, sample size was estimated using the double population proportion formula using OpenEpi online software, and the minimum sample size was far below the 911 obtained single population proportion formula and then 911 was considered as the so-called “minimum required sample size.” However, this study was part of a larger project on the impact of maternal common mental disorder on the obstetric outcome, birth outcomes, infant nutritional status and maternal functioning in Kersa and Haramaya Health and Demographic Surveillance Site, eastern Ethiopia, with a sample size of 1,160. Therefore, a total of 1,160 were considered as a total sample size.

**Figure 1:**
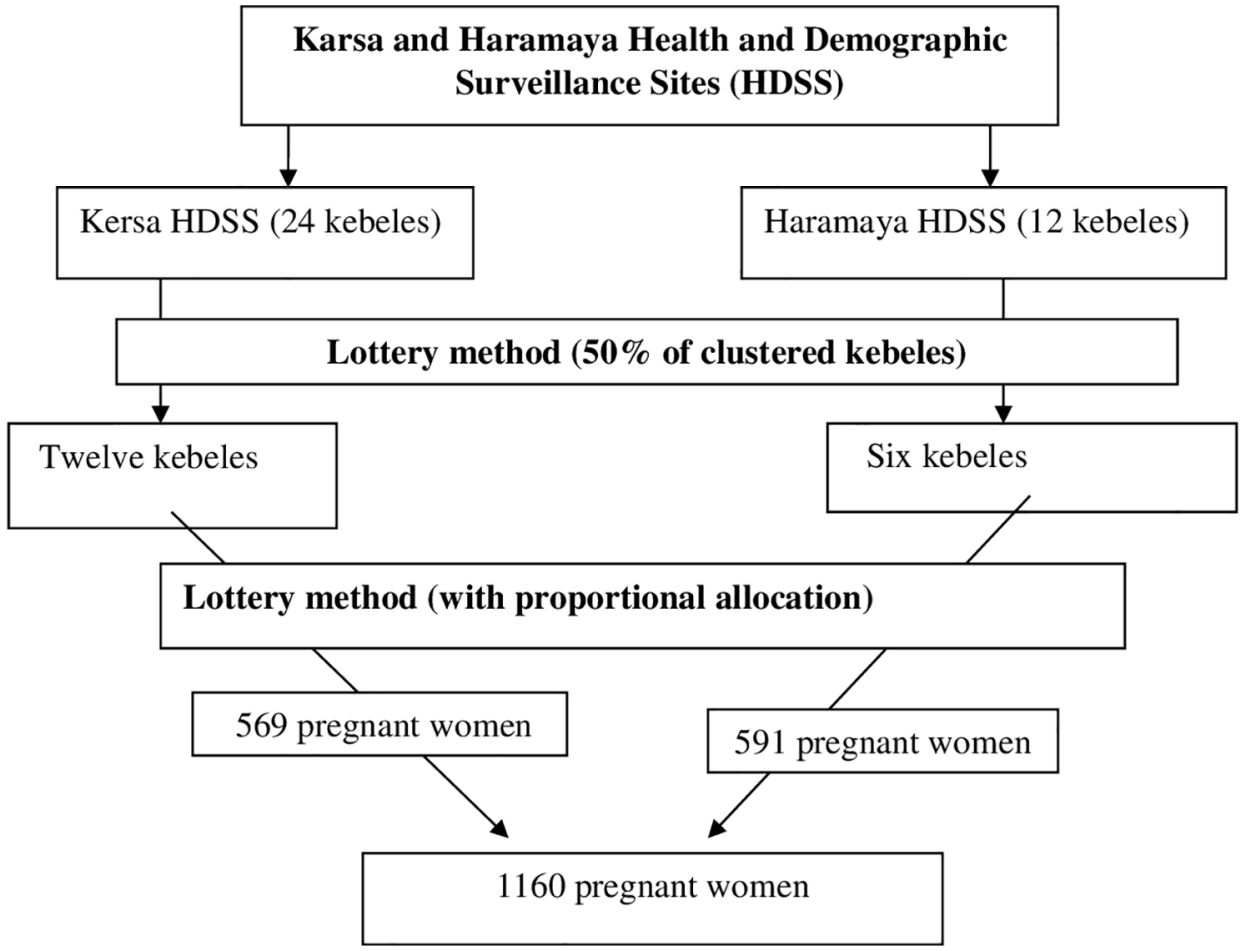
Schematic presentation of the sampling procedure for assessing khat use and associated factors among pregnant women.

There were 24 kebeles in Kersa and 12 kebeles in Haramaya HDSS sites. From the total kebeles on each site, sample kebeles were selected using lottery methods. Finally, the total sample size was proportionally allocated to the total population of pregnant women in sampled kebeles, and they were selected using lottery methods (Figure 1).

### Data collection procedure

Twenty data collectors who have been working in Kersa and Haramaya HDSS sites collected the data for this study. In addition, six MSc holders were supervising the data collection process. An intensive 3-day training was provided for data collectors and supervisors. The data were collected by face-to-face interviews using structured and semistructured questionnaires prepared in an Open Data Kit collect form, and completed data were sent directly to the server. Pregnant women were interviewed in separate rooms in their home environment or inside their compounds during all working hours of seven days a week. During the interviews, all the WHO recommendations/precautions for the prevention of COVID-19 infection were implemented.

### Data quality assurance

To maintain consistency of the data collection tool, the questionnaire was prepared first in the English language and translated to Afan Oromo and back to the English language by professional translators. Data collectors and supervisors took training. To evaluate the acceptability and applicability of the procedures and tools, a pretesting was carried out on 5% of the sample size in Harar town 1 week before the actual data collection. To maintain the completeness and consistency of the questionnaire, supervisors and investigators closely supervised the data collectors during the data collection process.

### Statistical analysis

After collection, the data clerk checked and cleaned the data; each questionnaire was checked for completeness and then coded. The data clerk entered the data into the computer using EpiData (Version 3.1, EpiData Association, Denmark) and then exported and analyzed the data using SPSS (Version 26; IBM Corp., Armonk, NY, United States). Categorical variables were described using frequencies and percentages.

In this study, bivariate and multivariate logistic regression was used. All variables with a *p*-value of ≤0.25 in bivariate logistic regression were taken to multivariable logistic regression. The strength of the association was described by odds ratio and 95% confidence interval (CI), and a *p*-value less than 0.05 was considered statistically significant. Multicollinearity of independent variables was tested using the variance inflation factor (VIF). In addition, model goodness of fit was checked by using Hosmer–Lemeshow test and the *p*-value was 0.834.

## Results

### Sociodemographic characteristics

From a total of 1,160 participants selected for the study, 1,015 consented to participate in the study yielding a response rate of 87.5%. Those participants who refused to participate in this study complained multiple reasons without any regard to the sociodemographic, clinical, and reproductive conditions. The mean age of the participants was 30.1 years (SD = 8.5). The majority of the study participants [982 (96.75%)] were married in their current relationship; 1,012 (99.7%) were Muslim followers and 1,010 (99.51%) were Oromo in ethnicity. About 772 (76.06%) of the participants neither read nor write and 900 (88.67) were housewives. About 388 (38.2%) participants were on the first quantile of the wealth index (Table 1).

**Table 1:** Sociodemographic and economic characteristics of pregnant women at Kersa and Haramaya HDSS sites, 2021

Variables	Category	Frequency (*N* = 1,015)	Percentage
Age	<25	94	9.26
	25–35	676	66.60
	≥35	245	24.14
Marital status	Married	982	96.75
	Others	33	3.25
Religion	Muslim	1,012	99.70
	Orthodox	3	0.30
Ethnicity	Oromo	1,010	99.51
	Amhara	5	0.49
Educational status	Literate	218	21.48
	Can read and write	25	2.46
	Neither read nor write	772	76.06
Occupation	Housewife	900	88.67
	Farmer	47	4.63
	Students	23	2.3
	Others	45	4.43
Wealth index	First quantile	388	38.23
	Second quantile	375	36.95
	Third quantile	252	24.83

HDSS, Health and Demographic Surveillance Sites.

### Clinical characteristics

**Figure 2:**
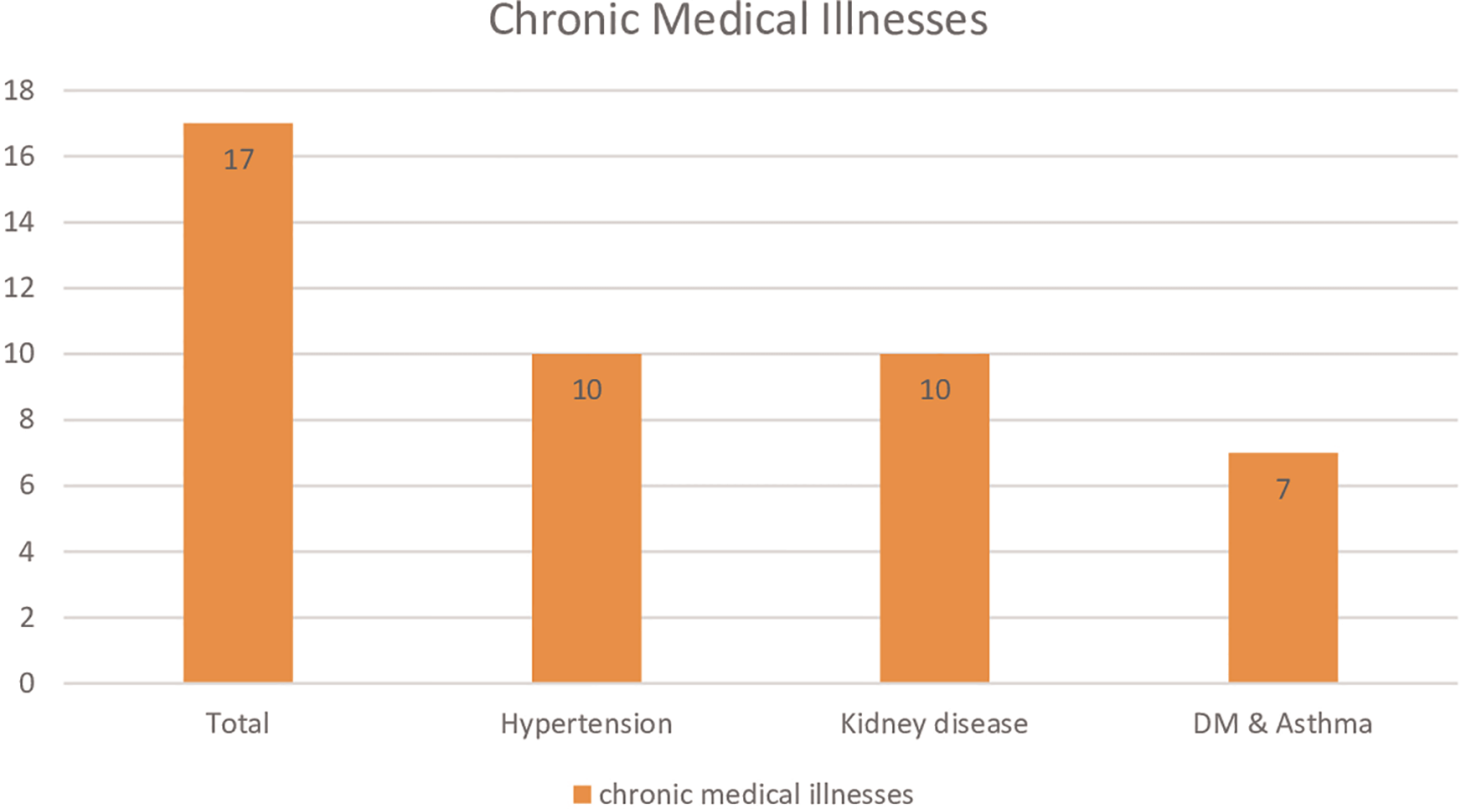
Types of chronic medical illnesses among pregnant women.

The study showed that 26 (2.56%) of the participants included in this study had a history of mental illness with a mean duration of 2.7 years (SD = 1.37). Of them, 10 (38.46%) were on treatment during the study period. On the other hand, 23 (2.27%) of the participants had chronic medical illnesses and 17 (73.91%) were on treatment. Hypertension [10 (43.48%)], kidney disease [10 (43.48%)], and other chronic illnesses such as diabetes and asthma [7 (30.34%)] were the medical conditions reported by the women (Figure 2).

### Reproductive, obstetric, and gynecological characteristics

The majority of the study participants were multigravida of 4 and above [542 (53.40%)]. About 472 (46.50%) of the participants were multipara of 4 and above. Over half (52.12%) of the participants were in the third trimester and the median gestational age of the pregnant women was 32 weeks (IQR = 8). Ninety-eight (9.66%) of the participants had a history of abortion, while 488 (48.08%) have antenatal care (ANC) follow-up. About 70 (6.9%) of the participants had a history of gynecological problems, where tumor (41.43%) was the most prevalent one. The mean pregnancy interval and age at first marriage were 1.7 years (SD = 0.56) and 17.7 years (SD = 2.12), respectively (Table 2).

**Table 2:** Reproductive, obstetric, and gynecological characteristics of pregnant women at Kersa and Haramaya HDSS sites, 2021 (*n* = 1015)

Variables	Categories	Frequency	Percentage
Gravidity	Primigravida	141	13.9
	Multigravida (II)	140	13.8
	Multigravida (III)	192	18.9
	Multigravida (IV) and above	542	53.4
Parity	Nulliparous	52	5.12
	Primipara	160	15.8
	Multipara (II)	155	15.3
	Multipara (III)	176	17.3
	Multipara (IV) and above	472	46.50
Gestational age (in weeks)	First trimester	31	3.05
	Second trimester	455	44.83
	Third trimester	529	52.12
History of abortion	Yes	98	9.66
	No	917	90.34
ANC follow-up	Yes	488	48.08
	No	527	51.92
Interval between last delivery and LNMP (months)	<24	754	92.29
	24–59	55	6.73
	>59	8	0.98
History of gynecological problems	Yes	70	6.90
	No	945	93.10
History of gynecological operation	Yes	46	4.53
	No	969	95.47

ANC, antenatal care; LNMP, last normal menstrual period; HDSS, Health and Demographic Surveillance Sites.

### Psychosocial and other substance use characteristics

A significant proportion of the pregnant women had experienced IPV during the current pregnancy. About 493 (48.6%) had reported that they had experienced the violence. Very few, four and nine, of the participants ever used alcohol and tobacco, respectively. Two (0.2%) and six (0.6%) of the pregnant women were current users of alcohol and tobacco, respectively (Table 3).

**Table 3:** Psychosocial and other substance use behavioral characteristics of pregnant women at Kersa and Haramaya HDSS sites, 2021 (*n* = 1,015).

Variables	Category	Frequency (*N* = 1,015)	Percentage
Intimate partner violence	Yes	493	48.6
	No	522	51.4
Ever alcohol use	Yes	4	0.4
	No	1,011	99.6
Ever tobacco use	Yes	9	0.9
	No	1,006	99.1
Current Alcohol use	Yes	2	0.2
	No	1,013	99.8
Current tobacco use	Yes	6	0.6
	No	1,009	99.4

HDSS, Health and Demographic Surveillance Sites.

### Prevalence of khat use

The result of this study showed that the prevalence of current khat use among pregnant women was 15.5% (95% CI, 13.3–17.7). The prevalence of ever khat use was 20.1% (95% CI, 17.8–22.5).

### Description of the outcome variable with some selected independent variable

About 154 of the married pregnant women use khat and 143 of the pregnant housewives were using khat. Sixty-four pregnant women from the second quartile of the wealth index use khat; 70 pregnant women who have experienced intimate partner violence use khat while 87 pregnant women who have not experienced intimate partner violence use khat. All (6) of the current tobacco user pregnant women were also using khat. About 114 pregnant women who have four or more pregnancies were using khat, about 98 pregnant women who have delivered four or more times use khat, and 5 pregnant women who had gynecological operations use khat (Table 4).

**Table 4:** Selected variables and the magnitude of khat use during pregnancy at Kersa and Haramaya HDSS sites, 2021 (*n* = 1,015).

S No.	Selected variables		Khat use during current pregnancy	
			Yes	No
1	Marital status	Married	154	851
		Others^a^	3	7
2	Occupational status	Housewife	143	757
		Farmer	4	43
		Students	4	19
		Others^b^	6	39
3	Wealth index	First quantile	58	330
		Second quantile	64	311
		Third quantile	35	217
4	Intimate partner violence	Yes	70	423
		No	87	435
5	Ever-use of tobacco	Yes	5	4
		No	152	854
6	Current use of tobacco	Yes	6	0
		No	151	858
7	Gravidity	Primigravida	7	134
		Multigravida (II)	21	119
		Multigravida (III)	15	177
		Multigravida (IV) and above	114	428
8	Parity	Nulliparous	4	48
		Primipara	18	142
		Multipara (II)	17	138
		Multipara (III)	20	156
		Multipara (IV) and above	98	374
9	Gynecological operations	Yes	5	41
		No	152	817

HDSS, Health and Demographic Surveillance Sites.

^a^ Separated, widowed, or divorced.

^b^ Government employer, non-government employer, petty vender, or unemployed.

### Factors associated with khat use during pregnancy

To identify different factors associated with khat use bivariate and multivariable logistic regressions were performed. In the bivariate logistic model age, educational status, presence of common mental disorder, presence of chronic medical illnesses, number of deliveries, history of abortion, ANC follow-ups, and gynecological problems were associated with khat use with a *p*-value of ≤0.25. However, after controlling the potential confounders by using multivariable logistic regression, only age, presence of medical illness, and history of abortion were significantly associated with khat use among pregnant women with a *p*-value of <0.05.

Those pregnant women aged 25–35 were 2.27 times more likely to use khat during the current pregnancy than those pregnant women aged less than 25 years old [adjusted odds ratio (AOR) 2.27, 95% CI, 1.33–4.89]. Likewise, those pregnant women aged 35 or more years were 2.33 times more likely to use khat as compared with those aged less than 25 years old (AOR: 2.33, 95% CI, 1.29–4.20). Additionally, those pregnant women with chronic medical illnesses were 3.28 times more likely to be khat users as compared with those pregnant women without chronic medical illnesses (AOR: 3.28, 95% CI, 1.27–8.48). Finally, those pregnant women with a history of abortion were 2.87 times more likely to be khat users during the current pregnancy than those without a history of abortion (AOR: 2.87, 95% CI, 1.73–4.76) (Table 5).

**Table 5:** Factors associated with khat use among pregnant women in Kersa and Haramaya HDSS sites, 2021.

Explanatory variables	Category	Khat use		OR (95% CI)		*p*-value
		Yes	No	COR (95% CI)	AOR (95% CI)	
Age	<25	22	262	1	1	
	25–35	85	401	2.5 (1.54–4.14)	**2.27** (1.33–4.89)**	**0**.**003**
	≥35	50	195	3.1 (1.79–5.21)	**2.33** (1.29–4.20)**	**0**.**005**
Educational status	Uneducated	112	660	1	1	
	Educated	45	198	1.34 (0.92–1.96)	0.7 (0.46–1.07)	0.096
CMD	No	85	549	1	1	
	Yes	72	309	1.51 (1.07–2.12)	1.15 (0.79–1.68)	0.46
Chronic medical illnesses	No	148	844	1		
	Yes	9	14	3.67 (1.56–8.62)	**3.28** (1.27–8.48)*	**0**.**014**
Number of deliveries	0	6	46	1	1	
	1	18	142	1.52 (0.49–4.72)	1.52 (0.47–4.95)	0.48
	2	17	155	1.48 (0.4–4.61)	1.38 (0.42–4.56)	0.59
	3	20	156	1.54 (0.50–4.72)	1.24 (0.38–4.03)	0.72
	≥4	98	374	3.14 (1.11–8.93)	2.05 (0.68–6.24)	0.21
History of abortion	No	120	797	1	1	
	Yes	37	61	4.03 (2.57–6.33)	**2.87** (1.73–4.76)***	**<0**.**001**
ANC follow-up	No	63	425	0.68 (0.48–0.97)	0.71 (0.50–1.03)	0.07
	Yes	94	433	1	1	
Gynecological problem	No	139	806	1	1	
	Yes	18	52	2.0 (1.14–3.53)	1.48 (0.79–2.76)	0.22

HDSS, Health and Demographic Surveillance Sites; OR, odds ratio; COR, crude odds ratio; CI, confidence interval; AOR, adjusted odds ratio; CMD, common mental disorders; ANC, antenatal care.

The bold values indicate the presence of statistically significant association.

* *p*-value < 0.05; **p-value < 0.01; ***p-value < 0.001.

## Discussion

The prevalence of khat use among pregnant women in this study was 15.5% (95% CI, 13.3–17.7). The finding of this study is higher than studies conducted in other parts of the country. According to the Ethiopian Demographic and Health Survey 2016, the current prevalence of khat chewing among women was 8.4% (16) and a study conducted in the Gedeo zone among pregnant women in primary healthcare revealed that the prevalence was 9.9% (15). The discrepancy might be attributable to variations in the characteristics of the sample, cultural differences in attitudes toward chewing a khat, and methodological differences between the studies. For instance, the study conducted in the Gedeo zone is a facility-based survey compared with our study which is a community-based study that involved a sample of mothers from rural areas, which is known to have variation from urban areas in both substances use patterns and access to healthcare (23, 24).

On other hand, this finding was lower than studies conducted in other parts of Ethiopia and other countries: in eastern Ethiopia (19.6%) (25), Butajira (35.8%) (14), Jimma (24.9%) (26), and Yemen (40.7%) (5). These variations might be due to the low concurrence use of other substances such as cigarettes and alcohol in the study samples. This pattern was different from other countries such as Yemen where simultaneous use of khat and tobacco is common, particularly among men (27). As evidenced by another study (28), social desirability of response bias, self-reporting about substance use could be the other reason for the lower prevalence of khat use.

The result of this study revealed that the age of the mothers, history of medical illness, and history of abortion were significantly associated with current khat use among pregnant women.

The odds of pregnant women aged 25–35 years were 2.39 times more likely to use khat when compared with those aged less than 25 years. The odds of those pregnant women aged greater than or equal to 35 years or more were 2.52 times more likely to use khat currently than those aged less than 25 years old. This indicates that as the age increased, the odds of khat use during pregnancy also increased. This result was congruent with a community-based study conducted in Ethiopia (16), and a national population survey conducted in Yemen where older women were shown to be khat chewers (5). The possible reason may be that as age increases, women are more likely to have changes in life situations such as bereavement, loneliness, poor social support, and financial difficulties, all of which have been found to increase the risk of substance use (29, 30).

The result of this study revealed another associated factor, and that those pregnant women who had a history of chronic medical illness were 3.18 times more likely to use khat during pregnancy than those who have had no history of medical illness. In a cross-sectional study of psychoactive substance use among pregnant women attending antenatal clinics at a federal government-owned tertiary hospital in Ogun state, southwestern Nigeria, there was an association between the presence of the medical conditions and lifetime use of other drugs (11). Also, it has been shown that in a study of 1,141 consecutive deliveries at delivery centers in the Yemen Arab Republic, significantly more khat chewers had concomitant diseases (31). The most likely explanation for this association is that khat has an identified wide range of adverse effects on the cardiovascular, gastrointestinal, and other peripheral systems (32–35).

Furthermore, in our study, pregnant women with a history of abortion had a higher odd of khat use. Those pregnant women who had a history of abortion were 2.79 times more likely to use khat during pregnancy than those who had no history of abortion. A study conducted in southern Ethiopia (36) revealed that there is a significant association between the history of abortion and substance use. Similarly, different studies conducted in the United States (9, 10, 37) and Norway (38) have shown a significant association between the history of abortion and substance use. There are several possible explanations for this significant finding. Women with a history of abortion reported five times more subsequent abuse of substance following abortion. Abortion can be considered one of life's stressful events, which leads women to use substances as a coping mechanism. On the other hand, pregnancy can also be considered stressful and may lead women to begin abusing substances (39). Abortion was found to be associated with an increased risk of substance abuse disorders (40). Women with a history of abortion may have a greater need to use emotion-altering substances during pregnancy, because the subsequent pregnancy may arouse unresolved feelings related to the abortion. Women with a history of abortion, compared with their peers who opt for delivery, also may be more liberal, inclined to take risks, and/or tend to be involved in difficult partner relationships more often (41, 42).

### Limitations of the study

This study has some limitations. Due to the nature of a cross-sectional study design, we could not explore the cause-and-effect relationships of the khat use and the independent variables. In addition to this, the study did not include the quantity and frequency of khat use. Furthermore, the laboratory screening for khat use was not performed, and this makes it difficult to corroborate the findings. Finally, a face-to-face interview method might induce recall bias and social desirability responses bias.

## Conclusions

The current study revealed that the use of khat was relatively high among pregnant women as compared with the previous studies. Special emphasis is paramount for pregnant women of increased age, those with medical illnesses, and those with a history of abortion, which were the factors significantly associated with khat chewing during pregnancy.

## Data Availability

The raw data supporting the conclusions of this article will be made available by the authors, without undue reservation.
